# Draft Genome Sequence of Raphidiopsis raciborskii Strain GIHE 2018, Isolated from a Shallow Freshwater Pond in South Korea

**DOI:** 10.1128/MRA.01545-19

**Published:** 2020-02-06

**Authors:** Ju-Yong Jeong, Sang-Hoon Lee, Mi-Ra Yun, Seung-Eun Oh, Chan-Won Hwang, Mi-Hye Yoon, Hee-Deung Park

**Affiliations:** a Department of Water Environment Research, Gyeonggi-do Institute of Health and Environment, Suwon, South Korea; b School of Civil, Environmental and Architectural Engineering, Korea University, Seoul, South Korea; University of Southern California

## Abstract

The draft genome sequence of Raphidiopsis (*Cylindrospermopsis*) raciborskii strain GIHE 2018, a filamentous nitrogen-fixing and potentially harmful cyanobacterium, is reported here. This is the first strain isolated from a shallow freshwater pond in South Korea. This information is expected to improve our understanding of the phylogeography of this species.

## ANNOUNCEMENT

Raphidiopsis raciborskii (formerly *Cylindrospermopsis raciborskii*) is a filamentous nitrogen-fixing and potentially harmful cyanobacterium originating in the tropics (1). The geographical range of *R. raciborskii* has expanded worldwide because of global climate change and its adaptability to various environments (2, 3). Despite morphological differences, this species was reclassified as belonging to the *Raphidiopsis* genus in 2018 based on polyphasic (4) and genetic marker-based analyses (5). *R. raciborskii* strain GIHE 2018 was observed and isolated in a shallow freshwater pond in South Korea for the first time (6).

A shallow freshwater pond sample was collected from Incheon, South Korea, using the grab sampling method (7). Strain GIHE 2018 was isolated using the micropipetting method (8) and cultured in Jaworski’s medium (9) at 25°C under a 16/8-h light/dark cycle. After extracting the total DNA using a water DNA purification kit (Norgen, Thorold, Canada), the PacBio (Menlo Park, CA) and Illumina (San Diego, CA) sequencing libraries were prepared using a P6 PacBio DNA/polymerase binding kit, a PacBio DNA sequencing kit 4.0, 8 single-molecule real-time (SMRT) cells, and the TruSeq library kit, respectively, according to the manufacturer's protocols. The genome was sequenced using one run each of the PacBio RS II and Illumina HiSeq 2500 platforms, respectively.

After the removal of adaptor and low-quality sequences by self-correction during the preassembly step using FALCON-integrate software v2.1.4 (10; https://github.com/PacificBiosciences/FALCON-integrate), 119,828 subreads (1,046,050,466 bp) were generated from the PacBio platform and used for the assembly step using the same software. From Illumina paired-end sequencing, 14,386,820 reads (100 ± 0 bp) were retrieved. The quality-filtered sequence reads from the Illumina sequencing, where 90% of bases had a Phred score of 30 or higher, were used for error correction using Pilon v1.21 (11; https://github.com/broadinstitute/pilon/wiki). The final coverage (depth) of the sequenced genome was 63×. Then, the genome was annotated using NCBI Prokaryotic Genome Annotation Pipeline (PGAP) v.4.8 (12, 13). Default parameters were used for all software unless otherwise specified.

From the PacBio sequencing, three uncircularized contigs were retrieved, with an *N*_50_ value of 1,278,585 bp (after assembly correction) and gaps of 33,630 bp (between contigs 1 and 2, gap = 21,674 bp; 2 and 3, gap = 11,956 bp) compared to those of the reference genome (GenBank accession number NZ_ACYA00000000.1). Alignment was performed to find matched areas between the draft genome and the reference genome using NUCmer (nucleotide MUMmer) software v4.0 (http://mummer.sourceforge.net/). After that, the gap size was calculated using Assemblytics Web-based software with the default option.

The genome information provided by PGAP showed that the genome contains 3,629,822 bp with 40.2% GC content and 3,265 coding sequences, 2,995 proteins, 270 pseudogenes, 42 tRNA genes, 9 rRNA genes, and 4 noncoding RNA (ncRNA) genes. Four CRISPR arrays were also discovered via PGAP. Moreover, four genes encoding a nonribosomal peptide synthetase complex related to hassallidin biosynthesis were identified.

Several strains of *Raphidiopsis* species produce toxins such as cylindrospermopsin or saxitoxin (14). However, in the GIHE 2018 genome, gene clusters associated with the biosynthesis of these cyanobacterial toxins were absent, suggesting that the isolated strain does not produce cyanobacterial toxins in its natural environment.

### Data availability.

The draft genome sequence of *Raphidiopsis raciborskii* GIHE 2018 has been deposited in NCBI under accession numbers VHLJ01000001 through VHLJ01000003 (GenBank), PRJNA548643 (BioProject), and SRR10071356 through SRR10071359 (SRA).
